# Natural Products in Endometrial Cancer: Molecular Mechanisms, Preclinical Evidence, and Clinical Perspectives

**DOI:** 10.3390/cimb48040408

**Published:** 2026-04-16

**Authors:** Hsien-Chang Wu, Chung-Che Tsai, Chan-Yen Kuo

**Affiliations:** 1 School of Post-Baccalaureate Chinese Medicine, Tzu Chi University, Hualien 970374, Taiwan; xuang@tzuchi.com.tw; 2 Department of Chinese Medicine, Taipei Tzu Chi Hospital, The Buddhist Tzu Chi Medical Foundation, No. 289, Jianguo Rd., Xindian Dist., New Taipei City 23142, Taiwan; 3 Department of Nursing, Cardinal Tien College of Healthcare and Management, New Taipei City 231038, Taiwan; 4 Department of Dentistry, Taipei Tzuchi Hospital, The Buddhist Tzu Chi Medical Foundation, New Taipei City 23142, Taiwan; 5 Institute of Oral Medicine and Materials, College of Medicine, Tzu Chi University, Hualien 970374, Taiwan

**Keywords:** endometrial cancer, natural product, phytochemicals, estrogen signaling, network pharmacology, integrative oncology

## Abstract

Endometrial cancer (EC) is the most common gynecologic malignancy in developed countries, with increasing incidence linked to obesity and metabolic dysfunction. While early-stage EC is often curable, advanced and recurrent disease remains difficult to treat due to resistance and limited therapeutic options. Natural products derived from traditional Chinese medicine have attracted attention as complementary strategies in EC management. These compounds exhibit multi-target effects, including modulation of estrogen signaling, inhibition of proliferation, induction of apoptosis, and regulation of immune and inflammatory pathways. This review summarizes current evidence on natural products in EC, integrating preclinical findings, emerging clinical data, and mechanistic insights from molecular and systems biology approaches. Key challenges, including variability, bioavailability, and insufficient clinical validation, are discussed. Future directions emphasize the integration of natural products into precision oncology frameworks.

## 1. Introduction

Endometrial cancer (EC) represents a major public health concern, particularly in high-income regions, where it accounts for the highest incidence among gynecologic malignancies [1]. According to global cancer statistics, both incidence and mortality rates of EC have steadily increased over the past decades, largely driven by rising obesity prevalence, sedentary lifestyles, insulin resistance, and prolonged estrogen exposure [2]. Clinically, EC has traditionally been classified into two major subtypes: type I (estrogen-dependent, endometrioid histology) and type II (estrogen-independent, non-endometrioid histologies, such as serous and clear cell carcinoma) [3]. Although the traditional dualistic classification of endometrial cancer has been refined by contemporary molecular classification systems, extensive epidemiological and clinical evidence indicates that estrogen-dependent signaling and metabolic dysregulation, particularly obesity, insulin resistance, and metabolic syndrome, remain central to endometrial carcinogenesis and disease progression [4].

Standard treatment for EC involves total hysterectomy with bilateral salpingo-oophorectomy, often combined with lymph node assessment [5]. Adjuvant radiotherapy, chemotherapy, hormonal therapy, and, more recently, targeted therapy and immunotherapy are selected according to stage and risk stratification [6]. A Surveillance, Epidemiology, and End Results (SEER)-based prognostic model for stage III EC demonstrated that the survival benefit of adjuvant radiotherapy varies across risk groups, supporting individualized postoperative treatment strategies [7,8]. However, advanced, recurrent, and metastatic EC continues to carry a poor prognosis. Furthermore, treatment-related toxicity, drug resistance, and limited therapeutic efficacy highlight the need for novel therapeutic strategies and supportive interventions.

**Figure 1:**
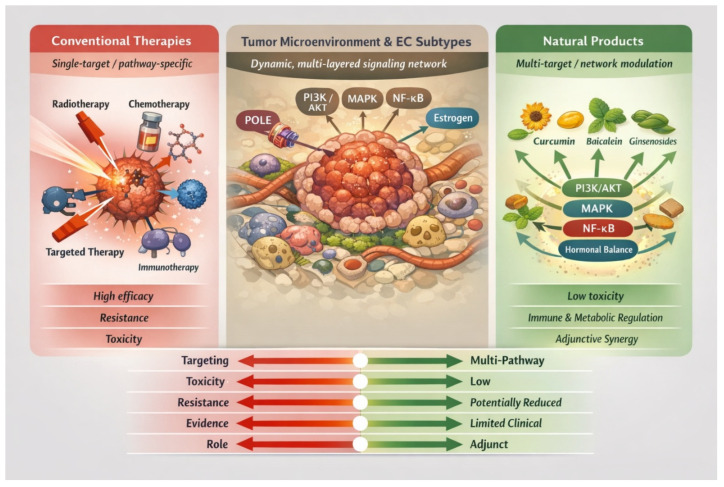
Mechanistic comparison between conventional adjuvant therapies and natural products in endometrial cancer. Conventional therapies achieve potent antitumor effects through target-specific mechanisms but are often limited by toxicity and resistance. In contrast, natural products exert multi-target, network-level modulation of oncogenic pathways and the tumor microenvironment, offering complementary, lower-toxicity benefits. This schematic illustrates the shift from reductionist, target-driven strategies to integrative, systems-based approaches in precision oncology.

Natural products offer a fundamentally different conceptual framework for disease prevention and treatment, emphasizing systemic balance, individualized therapy, and multi-component interventions [9]. Increasing interest in oncology research reflects its potential not only for symptom management and quality-of-life improvement but also for its potential direct anticancer effects [10]. This review aims to comprehensively evaluate the role of natural products in EC, focusing on biological mechanisms, experimental evidence, and clinical relevance. To highlight the conceptual differences between conventional adjuvant therapies and emerging natural product-based strategies, a systems-level comparison is illustrated in Figure 1. This framework emphasizes the shift from reductionist, single-target approaches toward multi-target network modulation in endometrial cancer.

## 2. Modern Biomedical Correlates of Transitional Chinese Medicine (TCM) Syndromes in EC

### 2.1. TCM Perspectives on EC: Syndrome Patterns, Qi–Blood Dysregulation, and Pathological Accumulation

In TCM, gynecologic malignancies are commonly characterized by phlegm-damp obstruction, liver-qi constraint, and blood stasis with “accumulation” (jie-ju). These patterns reflect impaired circulation of qi and blood and the formation of mass-like “accumulations” described in TCM terminology [11]. In addition, EC is interpreted within a TCM syndrome-based framework that emphasizes the coexistence of deficiency and excess patterns. Accordingly, therapeutic principles are focused on promoting blood circulation, resolving stasis, and eliminating pathogenic factors as part of individualized supportive management [12]. In summary, TCM conceptualizes gynecologic malignancies as a consequence of dysregulated qi–blood dynamics and pathological accumulation. This syndrome-based approach integrates deficiency and excess patterns and informs individualized strategies centered on restoring physiological balance while mitigating pathogenic factors.

### 2.2. Integrative Interpretation of TCM Concepts Within Modern Biomedical Frameworks

Modern biomedical research has identified parallels between TCM concepts and molecular pathophysiology. Modern biomedical research has identified meaningful parallels between TCM concepts and integrated biological systems. TCM syndrome patterns may reflect coordinated dysregulation of endocrine, immune, and inflammatory networks, aligning with systems biology perspectives. In particular, “liver-qi stagnation” can be interpreted as stress-related neuroendocrine–immune dysfunction. Chronic stress activates the hypothalamic–pituitary–adrenal axis and sympathetic signaling, leading to increased pro-inflammatory cytokines including interleukin-6 (IL-6) and tumor necrosis factor (TNF)-α, suppression of cytotoxic immune responses, and promotion of tumor-supportive microenvironments. These findings provide a mechanistic basis linking TCM theory with modern molecular pathophysiology [13].

In addition, recent evidence highlights the growing interest in natural products as potential therapeutic agents in endometrial cancer. These compounds exhibit multi-target activities, including modulation of cell proliferation, apoptosis, inflammation, and oxidative stress, thereby influencing key oncogenic signaling pathways. In addition to their direct anti-tumor effects, natural products may enhance treatment sensitivity and reduce adverse effects associated with conventional therapies, suggesting their potential role as complementary strategies in endometrial cancer management [14]. Liver-qi stagnation has been associated with stress-related neuroendocrine and immune dysregulation. Liver-qi stagnation, traditionally attributed to emotional dysregulation and impaired qi flow, can be mechanistically interpreted as a state of chronic stress induced neuroendocrine immune imbalance involving Hypothalamic–Pituitary–Adrenal (HPA) axis activation, inflammatory signaling, and systemic network dysfunction [15]. These integrative interpretations facilitate the translation of TCM theories into testable biomedical hypotheses and promote dialogue between traditional and modern frameworks.

## 3. Estrogen-Dependent Oncogenic Signaling Pathways as Therapeutic Targets in Endometrial Cance

### 3.1. Estrogen-Driven Carcinogenesis in EC: Molecular Mechanisms and Therapeutic Implications

Unopposed estrogen exposure is a central etiological driver of type I EC, particularly in the context of obesity, chronic anovulation, and prolonged estrogen replacement therapy [16]. Human males-absent on the first (MOF) (KAT8) is markedly upregulated in EC and serves as an independent prognostic factor. Its overexpression promotes tumor cell proliferation, migration, and invasion while inhibiting apoptosis. Estrogen–estrogen receptor (ER) signaling upregulates MOF expression and drives malignant phenotypes through activation of the phosphoinositide 3-kinase (PI3K)/protein kinase B (Akt) and rat sarcoma virus (Ras)/rapidly accelerated fibrosarcoma (Raf)/mitogen-activated protein kinase kinase (MEK)/extracellular signal-regulated kinase (ERK) pathways [17]. Beyond receptor activation, dysregulated estrogen biosynthesis and metabolism, characterized by increased peripheral aromatase activity in adipose tissue, altered hydroxylation pathways, and impaired estrogen clearance, further amplify the estrogenic burden within the endometrium [18]. EC is a hormone-driven malignancy in which estrogen signaling, mediated by ER-α, ER-β, and G protein-coupled ERs, regulates key cellular processes through diverse downstream pathways. Differential expression of ER-α splice variants and post-translational modifications further modulates these signaling cascades, underscoring their roles in endometrial carcinogenesis and their potential as targets for early diagnosis and therapeutic development [19]. TCM herbs, many of which contain bioactive phytoestrogens or exhibit anti-estrogenic properties, have emerged as potential modulators of estrogen-driven carcinogenesis [20].

### 3.2. Context-Dependent Dysregulation of PI3K/AKT/mammalian Target of Rapamycin (mTOR), ER-Mitogen-Activated Protein Kinase (MAPK), Wnt/β-Catenin, and Notch Signaling in EC Progression

Genomic analyses have identified frequent alterations in the PI3K/AKT/mTOR pathway in EC, promoting cell survival, proliferation, and metabolic reprogramming [21]. ER-α contributes to EC progression by regulating key co-expression modules and hub genes, primarily through activation of the MAPK signaling pathway [22]. Using physiologically relevant 3D spheroid models, MAPK14/p38α has been shown to function as a context-dependent regulator of high-grade EC, selectively driving stress adaptation, inflammatory signaling, metabolic reprogramming, and cancer stem–like cell maintenance—features not captured in conventional 2D cultures [23]. In addition, E2F transcription factor 8 (E2F8) promotes EC progression by transcriptionally upregulating denticleless E3 ubiquitin protein ligase homolog (DTL), leading to enhanced programmed cell death protein 4 (PDCD4) ubiquitination and subsequent MAPK activation [24]. Differential activation of MAPK cascades according to ER status further regulates proliferation and apoptosis, underscoring the importance of ER/MAPK crosstalk in disease progression and targeted intervention strategies [25]. Collectively, dysregulated PI3K/AKT/mTOR and ER-dependent MAPK signaling—coordinated in part by the E2F8/DTL/PDCD4 axis—represent central molecular drivers of EC progression and key targets for intervention.

Most sporadic ECs comprise endometrioid, serous, and clear cell histotypes, each exhibiting distinct clinical behavior and genomic profiles. Endometrioid tumors generally have a more favorable prognosis and frequently harbor PI3K and β-catenin pathway alterations, whereas serous and clear cell tumors are more aggressive and commonly exhibit TP53-associated genomic instability [26]. However, a molecular subset of endometrioid EC characterized by CTNNB1 exon 3 mutations demonstrates significantly reduced progression-free survival, highlighting the context-dependent role of aberrant β-catenin signaling and disrupted cell–cell adhesion in tumor progression [27]. Rowe et al. reported that nuclear β-catenin expression in EC, particularly in mismatch repair-deficient tumors, is associated with higher programmed death-ligand 1 (PD-L1) expression and may predict reduced responsiveness to immune checkpoint inhibitors [28]. Moreover, multi-drug resistance protein 4 (MRP4) stabilizes β-catenin and sustains Wnt/β-catenin signaling, contributing to both endometrial carcinogenesis and related pathologies [29]. Coordinated dysregulation of Wnt/β-catenin and estrogen signaling pathways, including crosstalk among ER-α, Dickkopf-1 (Dkk1), and β-catenin, further influences disease progression and clinical outcomes [30]. Although PTEN and β-catenin are frequently expressed in endometrioid EC, neither marker independently predicts overall or disease-free survival; however, phosphatase and tensin homolog (PTEN) positivity correlates with deeper myometrial invasion [31]. Together, these findings indicate that Wnt/β-catenin dysregulation drives EC progression through complex interactions with PI3K/PTEN, estrogen signaling, immune modulation, and adhesion pathways, with clinical relevance highly dependent on molecular context. Notch signaling also plays context-dependent roles in EC.

Aberrant Notch receptor and ligand expression suggests involvement in endometrial homeostasis and EC, although findings remain inconsistent [32]. In type I EC, progressive downregulation of Notch and Wnt pathways supports potential tumor-suppressive functions. Reduced MEG3 expression promotes tumor growth, whereas restoration suppresses proliferation through inhibition of the Notch1–hairy and enhancer of split-1 (Hes1) axis [33]. Conversely, Notch signaling maintains endometrial mesenchymal stromal/stem cell quiescence and regenerative capacity via interaction with Wnt/β-catenin signaling, while its inhibition impairs stem cell-driven endometrial repair in vivo [34]. Yokoi et al. reported that crosstalk between Notch effectors (Hes1/MAML2) and β-catenin signaling, driven by glycogen synthase kinase-3β (GSK-3β) inhibition, promotes β-catenin-mediated morular differentiation in EC and is associated with reduced proliferation and migration, suggesting a differentiation-linked, less aggressive tumor phenotype [35]. In contrast, CD133^+^ cancer stem-like cells exhibit activated NOTCH signaling, enhanced tumorigenicity, and resistance to epidermal growth factor receptor (EGFR) inhibition; dual targeting of *NOTCH* (DAPT) and *EGFR* (AG1478) synergistically suppresses tumor growth in vitro and in vivo, highlighting a promising combinational therapeutic strategy [36]. Dysregulated Wnt/β-catenin, Notch, and PI3K/AKT/mTOR pathways collectively contribute to therapy resistance and recurrence in EC, highlighting cancer stem cell-targeted strategies as promising therapeutic approaches [37]. In low-grade endometrial cancer, disruption of apicobasal polarity independent of E-cadherin loss impairs Notch receptor localization and signaling, thereby promoting reduced epithelial differentiation and enhanced tumor cell proliferation and migration, whereas restoration of Par3 reverses these effects in a Notch-dependent manner [38]. Qi et al. demonstrates that G protein-coupled estrogen receptor (GPER) is upregulated in endometrial cancer and promotes tumor cell proliferation and migration by mediating estrogen-driven activation of the NOTCH pathway. Both in vitro and in vivo findings indicate that estrogen enhances EC progression through a GPER/NOTCH1/Hes1 signaling axis, which can be attenuated by GPER suppression [39]. In summary, Notch signaling plays context-dependent roles in EC, functioning either as a tumor-promoting pathway through estrogen/GPER/NOTCH activation and cancer stem cell maintenance or as a tumor-suppressive/regenerative pathway via interactions with Wnt/β-catenin, cell polarity, differentiation, and stem cell quiescence. These divergent functions underscore Notch signaling as both a mechanistic driver and a potential biomarker or therapeutic target in endometrial cancer, depending on cellular context and tumor subtype. In addition, Notch signaling plays controversial, context-dependent oncogenic and tumor-suppressive roles in EC, influenced by cellular context, tumor subtype, and signaling interactions.

*TP53* mutations define the most aggressive subset of EC. These alterations remodel the tumor microenvironment, promote tumor progression, immune evasion, and are associated with poor prognosis [40]. The PORTEC-3 study demonstrated that p53 immunohistochemistry reliably reflects *TP53* mutation status using the WHO classification algorithm [41], and p53-defined molecular subtypes serve as strong prognostic markers for recurrence and poor disease-free survival [42]. p53-abnormal (p53abn) EC accounts for approximately 15% of cases but contributes disproportionately to disease-related mortality and includes both serous and non-serous histology’s with consistently poor outcomes [43]. p53abn expression is also observed in approximately 30% of FIGO grade 3 endometrioid EC and independently predicts poor overall and progression-free survival [44]. Reports of intratumoral heterogeneity, including spatially distinct p53-aberrant and p53-wild-type tumor components, further highlight molecular complexity [45]. *TP*53-mutated EC frequently exhibits human epidermal growth factor receptor 2 (HER2) overexpression or amplification, supporting HER2 as a therapeutic target [46]. Moreover, racial disparities in outcomes are closely linked to higher *TP53* mutation prevalence, and adjustment for molecular subtype eliminates race as an independent prognostic factor [47]. Non-hypermutant *TP53*-mutated tumors share molecular features across histological subtypes, supporting universal ERBB2 testing, although serous carcinomas remain clinically distinct due to more advanced presentation and worse disease-free survival [48]. p53 overexpression in EC independently predicts aggressive clinicopathologic features and poor overall survival, and only patients without p53 overexpression appear to derive significant survival benefit from adjuvant radiotherapy [49]. Collectively, *TP53* mutations characterize the most clinically aggressive EC subset by reshaping the tumor microenvironment and driving immune evasion, recurrence, and poor survival, with p53 immunohistochemistry serving as a reliable surrogate for *TP53* status and a robust prognostic and risk-stratification tool in clinical practice. Their association with HER2 alterations, intratumoral heterogeneity, racial survival disparities, and differential treatment response underscores *TP53* and related pathways as critical therapeutic targets. Table 1 summarizes the integration of EC molecular subtypes with potential TCM-based mechanistic targeting strategies. Collectively, dysregulated PI3K/AKT/mTOR, MAPK, Wnt/β-catenin, and Notch signaling pathways coordinated by estrogen receptor activity and reinforced by genetic alterations such as *TP53* mutations—constitute central molecular drivers of endometrial cancer progression, therapeutic resistance, and disease recurrence. These interconnected signaling networks provide critical targets for therapeutic intervention and form the mechanistic basis for current treatment strategies.

### 3.3. TCM as an Immunomodulatory Strategy in EC

EC represents a dynamic ecosystem in which tumor cells interact with diverse stromal and immune components within a remodeled extracellular matrix that shapes tumor initiation, progression, invasion, and metastasis [59]. Chronic inflammation and immunosuppressive conditions are common in EC, particularly in obesity-associated disease [60]. The immunomodulatory properties of TCM, including enhancement of antitumor immunity and attenuation of pro-inflammatory cytokine signaling, are increasingly recognized [61]. On the other hand, current management of EC is primarily based on a combination of surgical intervention and adjuvant therapies tailored to disease stage and molecular risk stratification. Total hysterectomy with bilateral salpingo-oophorectomy remains the cornerstone of treatment for early-stage disease, often achieving favorable outcomes [5]. Adjuvant radiotherapy provides effective locoregional control, particularly in high-risk patients, while systemic chemotherapy, typically platinum-based regimens, is employed in advanced or recurrent EC to reduce tumor burden and improve survival [6]. Hormonal therapies, including progestins and selective estrogen receptor modulators, are selectively applied in hormone receptor-positive tumors, offering a fertility-sparing or lower-toxicity alternative in carefully selected cases [6].

In recent years, the therapeutic landscape of EC has expanded with the integration of targeted therapies and immunotherapy. Agents targeting key oncogenic pathways, such as PI3K/AKT/mTOR and HER2, as well as anti-angiogenic therapies, have demonstrated clinical benefits in specific molecular subtypes [4,21]. Immune checkpoint inhibitors, particularly those targeting programmed cell death protein 1 (PD-1)/PD-L1, have shown promising efficacy in mismatch repair-deficient (MMR-d) and microsatellite instability-high (MSI-H) EC, as demonstrated in recent clinical trials [8,16]. These advances reflect the increasing importance of molecular classification in guiding treatment decisions and represent a shift toward precision oncology.

Despite these advances, conventional therapies remain constrained by several important limitations. Treatment-related toxicities, including hematologic, gastrointestinal, and cardiovascular adverse effects, can significantly impact patient quality of life and limit long-term use [6]. Moreover, intrinsic and acquired resistance to chemotherapy, targeted agents, and immunotherapy frequently leads to disease recurrence and progression [4]. Tumor heterogeneity and the complexity of signaling network crosstalk further challenge the effectiveness of single-target approaches. In this context, emerging strategies such as natural product-based interventions, characterized by multi-target and network-level modulation, may offer complementary benefits by enhancing therapeutic sensitivity, reducing toxicity, and addressing resistance mechanisms [10,14].

**Figure 2:**
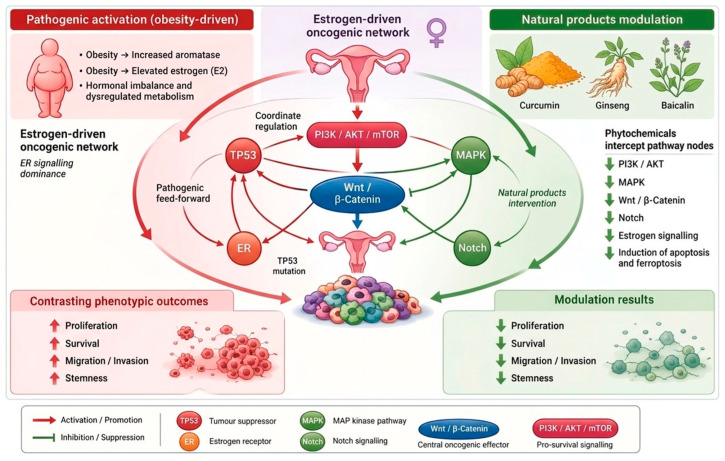
Systems-level comparison between obesity-driven pathogenic activation and natural product-mediated modulation in endometrial cancer. Obesity-associated estrogen excess drives ER signaling and metabolic dysregulation, activating key oncogenic pathways, including PI3K/AKT/mTOR, MAPK, Wnt/β-catenin, and Notch, with TP53 mutations further promoting tumor progression. In contrast, natural products such as curcumin, baicalein, and ginsenosides exert multi-target effects to suppress these pathways and restore cellular homeostasis, leading to induction of apoptosis and ferroptosis. Consequently, pathogenic signaling enhances proliferation, survival, invasion, and stemness, whereas natural product intervention attenuates these malignant phenotypes. Green-headed arrow indicates down-regulation, whereas red-headed arrow indicates up-regulation.

**Figure 3:**
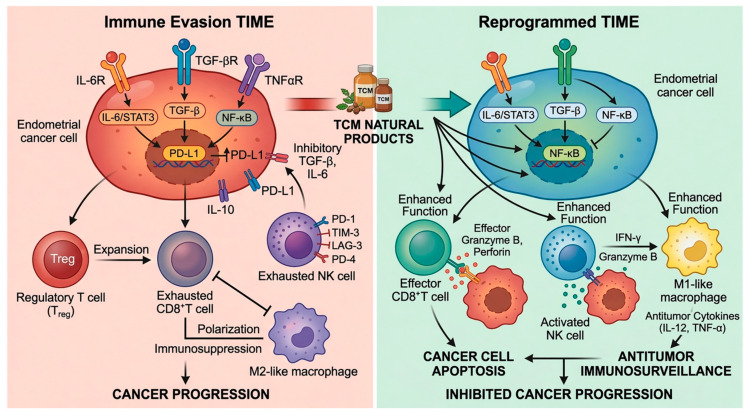
Immunomodulatory reprogramming of the tumor immune microenvironment (TIME) in endometrial cancer by TCM-derived natural products. The left panel illustrates an immunosuppressive TIME characterized by activation of the IL-6/STAT3, TGF-β, and NF-κB signaling axes in endometrial cancer cells, leading to upregulation of PD-L1 and secretion of immunosuppressive cytokines (e.g., IL-10). These events promote regulatory T cell (Treg) expansion, CD8^+^ T cell exhaustion, natural killer (NK) cell dysfunction (marked by PD-1, TIM-3, LAG-3, and PD-L1 interactions), and polarization of macrophages toward an M2-like phenotype, collectively facilitating immune evasion and tumor progression. The right panel depicts the reprogrammed TIME following intervention with traditional Chinese medicine (TCM)-derived natural products. These bioactive compounds suppress key oncogenic and immunosuppressive pathways (IL-6/STAT3, TGF-β, and NF-κB), thereby reducing PD-L1 expression and inhibitory cytokine signaling. Consequently, effector immune functions are restored, including enhanced cytotoxic activity of CD8^+^ T cells (granzyme B and perforin release) and NK cells (IFN-γ and granzyme B production), alongside repolarization of macrophages toward an M1-like phenotype that secretes antitumor cytokines (e.g., IL-12 and TNF-α). These coordinated immunomodulatory effects reinstate antitumor immunosurveillance, promote cancer cell apoptosis, and ultimately inhibit cancer progression.

Collectively, estrogen-driven signaling networks centered on ER-α, ER-β, and GPER coordinate key oncogenic pathways, including PI3K/AKT/mTOR, MAPK, Wnt/β-catenin, and Notch, thereby regulating proliferation, survival, invasion, and stemness in endometrial cancer. These effects are further reinforced by genetic alterations such as TP53 mutations, which contribute to disease progression and therapeutic resistance (Figure 2). Within this framework, natural products exert multi-target modulation of these interconnected pathways, providing a mechanistic rationale for their potential integration into complementary therapeutic strategies, as discussed in the following section. On the other hand, TCM-derived natural products exert immunomodulatory effects in endometrial cancer through multi-level regulation of immune cell populations, cytokine networks, and tumor microenvironment interactions, thereby restoring antitumor immunity and attenuating immune evasion. These immunomodulatory mechanisms are summarized in Figure 3. The tumor immune microenvironment in endometrial cancer is characterized by immunosuppressive features, including regulatory T cell expansion, macrophage polarization, and cytokine-driven signaling, which collectively contribute to tumor progression. Additionally, given these limitations and the increasing need for multi-target therapeutic approaches, growing attention has been directed toward natural products derived from traditional Chinese medicine. These agents exhibit broad-spectrum regulatory effects on oncogenic signaling pathways and the tumor microenvironment, providing a complementary framework to conventional therapies. The following section summarizes representative single herbs and bioactive compounds with demonstrated anti-endometrial cancer activity.

## 4. Single Herbs and Bioactive Compounds with Anti-EC Effects

### 4.1. Curcumin and Its Bioavailable Analogues as Multi-Target Therapeutic Agents in Endometrial Cancer

Curcumin, a polyphenolic compound derived from *Curcuma longa*, exhibits multifaceted anti-tumor effects in EC by inducing apoptosis, suppressing inflammation, inhibiting migration and invasion, and modulating key signaling pathways and microRNAs [53,62]. It inhibits proliferation and migration while inducing apoptosis and S-phase cell cycle arrest by suppressing ERK/c-Jun signaling through downregulation of ERK2 and Jun Proto-Oncogene (JUN) expression and reduced ERK and c-Jun phosphorylation [63]. Curcumin also inhibits migration and invasion of EC cells by suppressing ERK signaling and downregulating matrix metallopeptidase (MMP)-2/9 expression and activity [64]. Liposomal curcumin improves bioavailability and suppresses proliferation and motility while inducing apoptosis via NF-κB inhibition, with significant tumor suppression and minimal toxicity in zebrafish models [65]. Curcumin analogues HO-3867 and AKT-100 inhibit EC cell growth by inducing heme oxygenase 1 (HMOX1)-dependent ferroptosis and apoptosis, highlighting HMOX1 as a potential therapeutic target for overcoming drug resistance in advanced EC [55]. CP41, a bioavailability-enhanced curcumin derivative, exerts potent antitumor effects against EC by targeting H3F3A and inducing reactive oxygen species (ROS)-dependent MAPK activation, ER stress, and apoptosis with minimal in vivo toxicity [66]. Novel bioavailable curcumin analogues, AKT-100 and HO-3867, also restore wild-type tumor suppressor functions of mutant p53 in serous EC models by reinforcing cell cycle checkpoints, suppressing proliferative signaling, and promoting apoptosis [67]. A review further summarizes the broad anticancer effects of curcumin through modulation of Wnt/β-catenin, PI3K/Akt, MAPK, p53, and nuclear factor kappa-light-chain-enhancer of activated B cells (NF-κB) pathways and regulation of oncogenic and tumor-suppressive microRNAs [68]. Collectively, these findings position curcumin as a low-toxicity therapeutic agent that simultaneously targets multiple oncogenic signaling pathways and resistance mechanisms in advanced EC. Conversely, it represents a prototypical multi-target phytochemical capable of simultaneously modulating proliferative, inflammatory, and ferroptotic signaling cascades.

### 4.2. Scutellaria baicalensis and Flavonoids

Baicalein suppresses EC growth by activating DNA damage inducible transcript 4 (DDIT4)/AMP-activated protein kinase (AMPK) and inhibiting PI3K/mTOR signaling, and shows synergistic, low-toxicity antitumor effects when combined with metformin [57]. Proteomics analyses identified ADP-ribosylation factor 6 (ARF6) overexpression in EC cells; baicalein inhibits proliferation and invasion in a dose- and time-dependent manner by downregulating ARF6 and its downstream effectors Ras-related C3 botulinum toxin substrate 1 (Rac1) and p21 (RAC1) activated kinase 1 (PAK1), highlighting ARF6 suppression as a potential anticancer mechanism [69]. Wogonoside, a bioactive flavonoid derived from *Scutellaria baicalensis* Georgi, suppresses EC growth and metastasis by inducing ER stress and activating the Hippo signaling pathway via mammalian Sterile 20-Like Kinase 1 (MST1) phosphorylation [70]. *Scutellaria baicalensis* and *Fritillaria cirrhosa* inhibit ovarian and EC cell growth, anchorage-independent proliferation, and invasiveness at higher doses by inducing caspase-3 activation, G0/G1 arrest, cyclin D1/D3 downregulation, p27 induction, and NF-κB inhibition [54]. Collectively, flavonoids isolated from *Scutellaria baicalensis*, including baicalein and wogonoside, inhibit tumor growth and metastasis through coordinated suppression of PI3K/mTOR, ARF6/Rac1/PAK1, and NF-κB signaling pathways, while activating DDIT4/AMPK signaling, endoplasmic reticulum stress responses, and Hippo pathway regulation. These compounds demonstrate favorable safety profiles and synergistic interactions with metabolic modulators such as metformin.

### 4.3. Therapeutic Potential of Panax ginseng-Derived Ginsenosides in Endometrium-Related Diseases

*Panax ginseng* has long been used in East Asian traditional medicine as a restorative herbal remedy or adaptogen for individuals with chronic illnesses and is commonly included in medicinal formulations in China, Japan, and Korea, particularly in supportive cancer care [71]. Ginsenosides, the principal pharmacologically active constituents of ginseng, demonstrate therapeutic potential in endometrium-related disorders, including endometrial cancer, endometriosis, and endometritis, through coordinated regulation of cell death pathways, autophagy, epithelial–mesenchymal dynamics, immune modulation, and inflammatory signaling [51]. Moreover, Korean Red Ginseng (KRG) markedly attenuates Di-(2-ethylhexyl) phthalate (DEHP)-induced inflammatory responses by suppressing ERK1/2/NF-κB/cyclooxygenase-2 (COX-2) signaling in endometrial cancer Ishikawa cells and significantly reducing ectopic lesion growth in a mouse model of endometriosis [72]. 20(S)-Protopanaxadiol exerts anti-endometrial cancer activity by inhibiting tumor cell proliferation and xenograft growth through activation of caspase-dependent apoptosis, supporting its potential as a ginsenoside-derived scaffold for anticancer drug development [73]. In summary, *Panax ginseng*-derived ginsenosides, including Korean Red Ginseng and 20(S)-protopanaxadiol, demonstrate therapeutic potential in endometrium-related diseases by modulating inflammatory, immune, and cell death-associated signaling pathways, thereby suppressing disease progression in cellular and animal models. Ginsenosides exhibit both direct tumor-suppressive and microenvironment-regulatory effects.

### 4.4. Experimental Evidence Supporting the Anti-EC Effects of Astragalus membranaceus and Salvia miltiorrhiza Constituents

*Astragalus membranaceus* is a well-established herb in traditional Chinese medicine and is widely applied as an adjunctive therapeutic agent in the clinical management of multiple cancers [74]. An integrated network pharmacology analysis coupled with experimental validation revealed that *Astragalus membranaceus* exerts anti-endometrial cancer effects through multiple active compounds targeting p53-related, cell cycle, and transcriptional pathways, with formononetin inhibiting tumor cell proliferation by upregulating ER-β and p53 [52]. Salvianolic acid A suppresses endometrial carcinoma progression by inhibiting CD40-mediated AKT/mTOR/NF-κB signaling, thereby reducing tumor cell proliferation, invasion, and tumor growth, and inducing apoptosis and G0/G1 cell-cycle arrest in vitro and in vivo [75]. Dihydroisotanshinone I from Danshen inhibits endometrial cancer cell viability by inducing glutathione peroxidase 4 (GPX4)-dependent apoptosis and ferroptosis and effectively suppresses tumor growth in vivo without evident toxicity [56]. Together, *Salvia miltiorrhiza*-derived constituents exert potent anti-EC effects by disrupting key survival pathways and oxidative balance, leading to impaired proliferation, enhanced programmed cell death, increased ferroptotic susceptibility, and marked tumor suppression in experimental models.

### 4.5. Integrative Signaling Network Perspective

Although individual phytochemicals exhibit distinct molecular targets, evidence indicates convergence on central oncogenic hubs, including PI3K/AKT/mTOR, MAPK, NF-κB, estrogen-related signaling, and ferroptosis-associated oxidative pathways. Rather than functioning as single-target inhibitors, these compounds exert coordinated, multi-level network modulation. Table 2 summarizes the pathway-specific and cross-pathway regulatory profiles of representative TCM-derived phytochemicals in EC.

## 5. Bridging Preclinical and Clinical Evidence: Natural Compounds and Herbal Injections in Endometrial Cancer

Saikosaponin D demonstrates selective anti-EC activity by inducing G2/M cell-cycle arrest, activating death receptor– and mitochondria-mediated apoptosis, and suppressing migration and invasion through modulation of MAPK signaling, with minimal toxicity to normal cells [76]. A network meta-analysis of 25 randomized controlled trials involving 2023 patients indicates that combining Chinese herbal injections with chemotherapy significantly improves clinical efficacy, performance status, immune function, and safety outcomes in EC compared with chemotherapy alone. Among the evaluated regimens, Kangai, Aidi, and Eshuyou injections appear to offer the most favorable overall benefits, although further high-quality trials are needed to confirm these findings [77]. In summary, current evidence suggests that both isolated bioactive compounds and clinically applied herbal formulations hold therapeutic potential in endometrial cancer. Saikosaponin D exhibits multi-target antitumor activity with a favorable safety profile in preclinical models, while Chinese herbal injections, particularly Kangai, Aidi, and Eshuyou, may enhance the efficacy and tolerability of conventional chemotherapy in clinical settings. Nevertheless, these findings should be interpreted with caution due to heterogeneity in study design and variable methodological quality. Future well-designed, large-scale randomized trials and mechanistic studies are essential to validate efficacy, clarify molecular targets, and support their integration into evidence-based oncology practice. Table 3 summarizes the current hierarchy and strength of experimental and clinical validation supporting natural product interventions in EC.

Evidence hierarchy of TCM interventions in EC based on experimental level and clinical validation.

## 6. Systems Biology and Network Pharmacology Approaches

A network pharmacology strategy elucidates the multi-target, multi-pathway anticancer mechanisms of key phytochemicals, demonstrating coordinated regulation of apoptosis, cell-cycle control, oxidative stress, and major oncogenic pathways such as PI3K/Akt, MAPK, and p53 [78]. By integrating network pharmacology with molecular docking analyses, this study systematically elucidates the potential molecular mechanisms by which resveratrol inhibits type I EC, highlighting its regulation of key oncogenic, inflammatory, apoptotic, and angiogenic pathways, including PI3K/AKT, MAPK, and STAT3 signaling. In silico docking further demonstrates high binding affinity between resveratrol and central hub proteins such as MAPK3, MAPK8, and TNF, supporting its role as a multi-target therapeutic candidate and providing a rationale for further experimental validation [58]. Quercetin suppresses EC cell proliferation, invasion, and migration while promoting apoptosis by activating autophagy through inhibition of the ATF5/JUN/PI3K/AKT/mTOR signaling pathway, as demonstrated by network pharmacology and cellular validation in Ishikawa and HEC-1A cells [79]. Using network pharmacology, bioinformatics, and molecular docking analyses, Cheng et al. demonstrates that matrine may exert anti-uterine corpus endometrial carcinoma (UCEC) effects primarily through targeting cadherin 1 (CDH1) and epithelial cell adhesion molecule (EPCAM), two tumor-associated hub genes closely linked to immune infiltration and cancer progression [80]. In addition, Salidroside may act as a promising natural anti-EC agent by targeting multiple key molecules including AKT1, EGFR, caspase 3 (CASP3), hypoxia-inducible factor 1 subunit alpha (HIF1A), and MMP9 and modulating apoptosis- and PI3K/AKT-related signaling pathways, as demonstrated by integrated network pharmacology and molecular docking analyses [81]. Gambogic acid, a natural caged xanthone, exerts anti-EC effects by suppressing tumor growth through PI3K/AKT pathway inhibition, as revealed by network pharmacology analysis and validated experimentally via induction of G0/G1 cell-cycle arrest and mitochondria-dependent apoptosis with minimal toxicity [82]. Similarly, quercetin suppresses endometrial cancer progression by inhibiting the ATF5/JUN/PI3K/AKT/mTOR pathway and activating autophagy, thereby reducing proliferation, migration, and invasion while promoting apoptosis [79]. An integrated network pharmacology, metabolomics, molecular docking, and experimental approach reveals that maackiain suppresses EC progression by inducing G2/M cell-cycle arrest, altering tumor-associated metabolic pathways, and targeting key regulators including PLA2G10, PDE4D, and PDE5A [83]. Integrated network pharmacology, molecular docking, and experimental validation reveal that *Artemisia annua*, particularly its flavonoid quercetin, suppresses EC cell proliferation and migration by targeting and downregulating PTGS2, highlighting its multi-component therapeutic potential [84]. Echinacoside exerts significant anti-EC effects by targeting multiple genes and pathways identified through network pharmacology, with PI3K/AKT signaling emerging as a central hub. Experimental validation demonstrated that echinacoside inhibits proliferation and induces G2/M arrest, oxidative stress, mitochondrial dysfunction, and apoptosis in EC cells via suppression of the PI3K/AKT pathway [85]. Collectively, integrated network pharmacology analyses indicate that diverse phytochemicals exert anti-EC effects through coordinated, multi-target regulation of apoptosis, cell-cycle progression, oxidative stress, metabolism, immune-related hubs, and key oncogenic pathways, particularly PI3K/AKT, MAPK, p53, and signal transducer and activator of transcription 3 (STAT3). Molecular docking and experimental validation further support their potential as multi-pathway therapeutic candidates, providing a systematic mechanistic rationale for their potential in endometrial cancer intervention.

**Figure 4:**
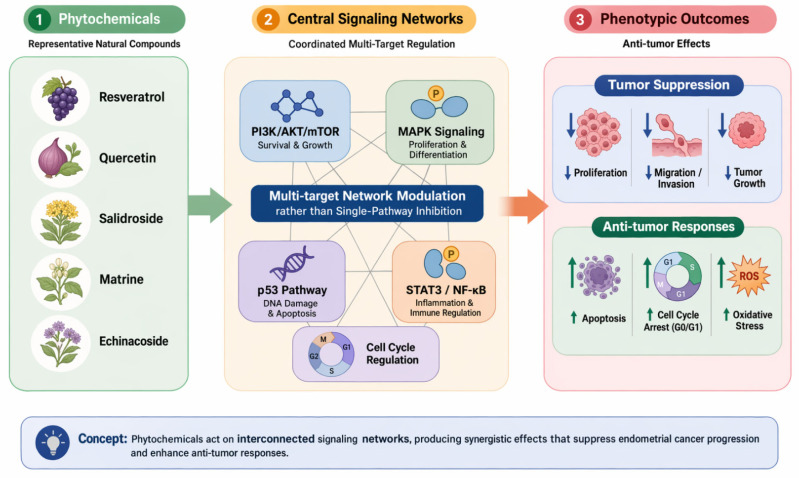
Network pharmacology–based multi-target mechanisms of natural products in endometrial cancer. This figure illustrates how phytochemicals (e.g., resveratrol, quercetin, salidroside, matrine, and echinacoside) exert anti–endometrial cancer effects through multi-target interactions identified by network pharmacology and molecular docking. These compounds converge on key hub proteins (e.g., AKT1, EGFR, MAPKs, TNF, CASP3, HIF1A) and modulate major pathways, including PI3K/AKT/mTOR, MAPK, STAT3, and p53 signaling. Coordinated pathway regulation leads to suppression of proliferation, migration, and angiogenesis, alongside induction of apoptosis, autophagy, oxidative stress, and cell-cycle arrest. Integrated computational and experimental validation supports a systems-level, network-based therapeutic strategy for precision oncology. Blue-headed arrow indicates down-regulation, whereas green-headed arrow indicates up-regulation.

Collectively, the integrative framework depicted in Figure 4 underscores the systems-level nature of natural product activity in endometrial cancer, whereby multi-component phytochemicals simultaneously target interconnected molecular networks rather than isolated pathways. By linking compound–target interactions with pathway modulation and phenotypic outcomes, and by integrating computational predictions with experimental validation, this model provides a robust mechanistic foundation for the development of network-based therapeutic strategies and supports the translation of natural products into precision oncology.

## 7. Challenges and Future Perspectives

Despite increasing preclinical, translational, and emerging clinical evidence supporting the anticancer potential of TCM in EC, several challenges limit its broader clinical adoption and mechanistic validation [10,12,77,86]. First, heterogeneity and lack of standardization remain major obstacles [87]. The chemical composition of herbal formulas and single herbs can vary substantially due to differences in botanical origin, cultivation conditions, harvesting time, and processing methods, resulting in batch variability and inconsistent pharmacological effects. Establishing standardized extraction procedures, robust quality control systems, and chemical fingerprinting strategies is therefore essential to ensure reproducibility and clinical reliability [88]. Second, pharmacokinetic complexity and bioavailability issues pose additional challenges. Many bioactive phytochemicals suffer from low aqueous solubility, rapid metabolic clearance, and insufficient systemic bioavailability, which can substantially limit their therapeutic effectiveness in vivo despite promising in vitro activity [89,90]. Advanced drug delivery systems, structural optimization, and formulation strategies such as nanoformulations or prodrug approaches represent promising avenues to enhance bioavailability and therapeutic index [91]. Third, high-quality clinical evidence remains insufficient. Although observational studies, cohort analyses, and network meta-analyses suggest potential benefits particularly as adjuncts to conventional therapy large-scale, randomized, double-blind, placebo-controlled trials specifically designed for EC are lacking [12,77]. Future clinical studies should incorporate rigorous design, clearly defined endpoints (e.g., survival, recurrence, and quality of life), and molecular stratification based on contemporary EC classifications, as emphasized in current clinical guidelines and recent translational research [50,92]. Fourth, mechanistic complexity and context dependency complicate interpretation. TCM interventions typically involve multi-component, multi-target interactions affecting signaling pathways, metabolic states, immune landscapes, and tumor microenvironmental contexts [93,94]. While systems biology and network pharmacology provide insights, computational predictions require deeper experimental validation using physiologically relevant models, such as patient-derived organoids, 3D cultures, and immunocompetent animal models [95,96]. Integration with precision medicine represents a key future direction. Aligning TCM interventions with molecular subtypes of EC, metabolic profiles, immune status, and patient-specific characteristics may enable more rational, personalized integrative strategies [97]. Moreover, combination approaches pairing selected TCM formulations or phytochemicals with targeted therapies, hormonal agents, or immunotherapies may help overcome resistance, reduce toxicity, and enhance efficacy [12,98,99]. In summary, advancing TCM from complementary use to evidence-based integration in EC management will require interdisciplinary collaboration among clinicians, pharmacologists, molecular biologists, and data scientists. Addressing standardization, clinical validation, and mechanistic clarity may facilitate its incorporation into integrative and precision oncology. Despite promising mechanistic and preclinical data, translational implementation requires careful consideration of pharmacokinetics, safety profiles, herb–drug interactions, and standardization challenges. Table 4 summarizes the comparative risk–benefit and clinical readiness of major TCM-derived agents discussed in this review. On the other hand, although this review primarily focuses on plant-derived natural products, we acknowledge that commercially available agents and clinically approved anticancer drugs derived from microorganisms, including fungi, algae, actinomycetes, and bacteria, constitute an important and expanding field. Due to space constraints, these aspects are not comprehensively discussed here but warrant dedicated investigation in future studies.

## 8. Summary

**Figure 5:**
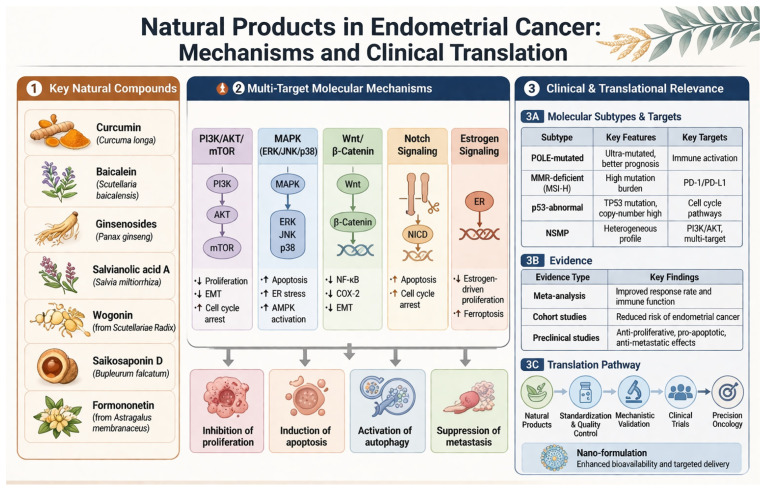
Natural products in endometrial cancer: molecular mechanisms and clinical translation. Key bioactive compounds, including curcumin, baicalein, ginsenosides, salvianolic acid A, wogonin, saikosaponin D, and formononetin, exert antitumor effects through multi-target modulation of major oncogenic pathways such as PI3K/AKT/mTOR, MAPK, Wnt/β-catenin, Notch, and estrogen signaling. These coordinated regulatory effects lead to inhibition of proliferation, induction of apoptosis, activation of autophagy, and suppression of metastasis. Emerging evidence from preclinical and clinical studies supports their therapeutic potential across molecular subtypes of endometrial cancer. Future translation requires standardization, mechanistic validation, and integration into precision oncology frameworks. AKT, protein kinase B; AMPK, AMP-activated protein kinase; COX-2, cyclooxygenase-2; EMT, epithelial–mesenchymal transition; ER, estrogen receptor; ERK, extracellular signal-regulated kinase; JNK, c-Jun N-terminal kinase; mTOR, mechanistic target of rapamycin; MSI-H, microsatellite instability-high; NICD, Notch intracellular domain; NF-κB, nuclear factor kappa B; PI3K, phosphoinositide 3-kinase. ↓: Down-regulation; ↑: Up-regulation.

EC is a molecularly heterogeneous malignancy characterized by dysregulated estrogen signaling, metabolic alterations, and recurrent genomic events, including PI3K/AKT/mTOR pathway activation and TP53 mutations. Although current therapeutic strategies have improved outcomes in early-stage disease, advanced and recurrent EC remains associated with limited efficacy and treatment resistance. Natural products have emerged as potential complementary agents with multi-target regulatory properties. Preclinical evidence indicates that these compounds modulate key signaling pathways, including MAPK, Wnt/β-catenin, Notch, NF-κB, and estrogen receptor-related networks, thereby affecting proliferation, apoptosis, metastasis, and tumor–microenvironment interactions. Systems biology and network pharmacology analyses further support their role in coordinated regulation of interconnected signaling pathways (Figure 5). However, translational applications are limited by variability in composition, pharmacokinetic constraints, and insufficient high-quality clinical evidence. Future studies should emphasize standardization, mechanistic validation in physiologically relevant models, and clinical investigation within molecularly stratified EC populations to support evidence-based integration into oncology practice.

## 9. Conclusions

Endometrial cancer (EC) is driven by complex interactions among hormonal, metabolic, and molecular factors, which limit the effectiveness of single-target therapeutic strategies. Evidence summarized in this review indicates that natural products exert multi-target regulatory effects on key oncogenic pathways, including PI3K/AKT/mTOR, MAPK, Wnt/β-catenin, Notch, NF-κB, and estrogen receptor signaling. These compounds influence tumor cell proliferation, apoptosis, metastasis, and tumor–microenvironment interactions in preclinical models, supporting their potential as complementary therapeutic agents. However, clinical translation remains constrained by variability in composition, limited bioavailability, potential herb–drug interactions, and insufficient high-quality clinical evidence. Future research should prioritize standardized formulations, rigorous pharmacokinetic evaluation, and well-designed clinical trials. Integration of natural products with molecular classification and precision oncology approaches may facilitate their rational application and improve therapeutic outcomes in EC.

## Figures and Tables

**Table 1:** Molecular Subtype-Driven Therapeutic Targeting Framework Linking Endometrial Cancer Genomics with Traditional Chinese Medicine Strategies.

Molecular Subtype	Key Genetic Features	Dominant Pathways	Clinical Behavior	TCM Mechanistic Rationale	Representative Agents	Key References
POLE-ultramutated	*POLE* mutation	DNA repair, immune activation	Excellent prognosis	Immune modulation & TME balance	Ginsenosides, Astragalus	[4,50,51,52]
MMR-deficient (MSI-high)	MLH1/MSH2 loss	PD-L1, inflammation	Intermediate	Anti-inflammatory & immune regulatory	Curcumin, Scutellaria	[4,28,53,54]
p53-abnormal	*TP53* mutation, *HER2* amplification	Cell cycle, ER stress	Poor prognosis	Apoptosis & ferroptosis induction	HO-3867, DHI	[43,46,55,56]
NSMP	PTEN, CTNNB1	PI3K/AKT, Wnt	Variable	Multi-pathway suppression	Resveratrol, Baicalein	[21,27,57,58]

**Table 2:** Multi-Target Signaling Network Modulation by Major TCM-Derived Phytochemicals in Endometrial Cancer.

Compound	PI3K/AKT	MAPK	Wnt	NF-κB	p53	Ferroptosis	Model	References
Curcumin	↓	↓ ERK	↓	↓	↑	↑ HMOX1	Cell, zebrafish	[55,63,65,68]
Baicalein	↓ mTOR	—	—	↓	—	—	In vitro	[57,69]
Wogonoside	—	—	—	—	—	—	Cell	[70]
Ginsenosides	↓	↓ ERK	—	↓ COX-2	—	—	Cell + mouse	[51,72,73]
Salvianolic Acid A	↓ AKT	—	—	↓ CD40/NF-κB	—	—	Cell + xenograft	[75]
Dihydroisotanshinone I	—	—	—	—	—	↑ GPX4-related	Cell + mouse	[56]

Signaling pathways modulated by representative phytochemicals. Arrows indicate inhibitory (↓) or activating (↑) effects supported by cited experimental studies.

**Table 3:** Stratification of Preclinical and Clinical Evidence Supporting Natural Products in Endometrial Cancer.

Intervention	Model	Mechanism	Key Outcome	Clinical Evidence	References
Curcumin	Cell + zebrafish	MAPK/NF-κB	Apoptosis, ↓ migration	None	[53,55,62,63,64,65]
Saikosaponin D	Cell	MAPK	G2/M arrest	None	[76]

Arrows indicate inhibitory (↓) effect supported by cited experimental studies.

**Table 4:** Risk–Benefit and Translational Feasibility of Selected TCM-Derived Agents in Endometrial Cancer.

Compound	Bioavailability	Toxicity	Interaction Risk	Combination Potential	References
Curcumin	Low (nano/liposomal improved)	Minimal	Low	Chemo, ICI	[65,91]
Baicalein	Moderate	Low	Limited data	Metformin	[57]
Ginsenosides	Good	Low	Possible CYP modulation	Immunotherapy	[51,72]
Salvianolic Acid A	Moderate	Low	Possible anticoagulant	Targeted therapy	[75]
Dihydroisotanshinone I	Emerging data	Low (animal)	Unknown	Resistant subtype	[56]

## Data Availability

No new data were created or analyzed in this study. Data sharing is not applicable to this article.

## Abbreviations

AKT	Protein Kinase B
AMPK	AMP-Activated Protein Kinase
ARF6	ADP-Ribosylation Factor 6
ATF5	Activating Transcription Factor 5
BSYX	Bu Shen Yang Xue
CA125	Cancer Antigen 125
CASP3	Caspase 3
CD40	Cluster of Differentiation 40
CDH1	Cadherin 1
COX-2	Cyclooxygenase-2
CTNNB1	Catenin Beta 1
CYP	Cytochrome P450
DDIT4	DNA Damage Inducible Transcript 4
DEHP	Di-(2-ethylhexyl) Phthalate
DHI	Dihydroisotanshinone I
DKK1	Dickkopf-1
DLT	Denticleless E3 Ubiquitin Protein Ligase Homolog
E2F8	E2F Transcription Factor 8
EC	Endometrial Cancer
EGFR	Epidermal Growth Factor Receptor
EPCAM	Epithelial Cell Adhesion Molecule
ER	Estrogen Receptor
ERK	Extracellular Signal-Regulated Kinase
ER-α	Estrogen Receptor Alpha
ER-β	Estrogen Receptor Beta
ESGO	European Society of Gynaecological Oncology
ESTRO	European Society for Radiotherapy and Oncology
FSH	Follicle-Stimulating Hormone
FIGO	International Federation of Gynecology and Obstetrics
GPER	G Protein-Coupled Estrogen Receptor
GPX4	Glutathione Peroxidase 4
GSK-3β	Glycogen Synthase Kinase-3β
HE4	Human Epididymis Protein 4
HES1	Hairy and Enhancer of Split-1
HER2	Human Epidal Growth Factor Receptor 2
HIF-α	Hypoxia-Inducible Factor Alpha
HIF1A	Hypoxia-Inducible Factor 1 Subunit Alpha
HMOX1	Heme Oxygenase 1
HSD17B1	Hydroxysteroid 17-Beta Dehydrogenase 1
ICI	Immune Checkpoint Inhibitor
IL-6	Interleukin-6
JUN	Jun Proto-Oncogene
KRG	Korean Red Ginseng
MAPK	Mitogen-Activated Protein Kinase
MEG3	Maternally Expressed Gene 3
MEK	Mitogen-activated Protein Kinase Kinase
MMP	Matrix Metalloproteinase
MMR	Mismatch Repair
MOF	Male Absent on the First
MSI	Microsatellite Instability
MST1	Mammalian Sterile 20-Like Kinase 1
mTOR	Mammalian Target of Rapamycin
NF-κB	Nuclear Factor kappa-light-chain-enhancer of activated B cells
NSMP	No Specific Molecular Profile
PAK1	p21 (RAC1) Activated Kinase 1
PDCD4	Programmed Cell Death Protein 4
PD-1	Programmed Cell Death Protein 1
PD-L1	Programmed Death-Ligand 1
PI3K	Phosphoinositide 3-Kinase
PLA2G10	Phospholipase A2 Group X
POLE	DNA Polymerase Epsilon
PORTEC	Post Operative Radiation Therapy in Endometrial Carcinoma
PTEN	Phosphatase and Tensin Homolog
Rac1	Ras-related C3 botulinum toxin substrate 1
Raf	Rapidly Accelerated Fibrosarcom
Ras	Rat Sarcoma Virus
ROS	Reactive Oxygen Species
RCT	Randomized Controlled Trial
SEER	Surveillance, Epidemiology, and End Results
SDF-1	Stromal Cell-Derived Factor 1
STAT3	Signal Transducer and Activator of Transcription 3
TCGA	The Cancer Genome Atlas
TCM	Traditional Chinese Medicine
TNF	Tumor Necrosis Factor
TP53	Tumor Protein p53
UCEC	Uterine Corpus Endometrial Carcinoma
Wnt	Wingless-Related Integration Site
